# Overview of nanoscale NEXAFS performed with soft X-ray microscopes

**DOI:** 10.3762/bjnano.6.61

**Published:** 2015-02-27

**Authors:** Peter Guttmann, Carla Bittencourt

**Affiliations:** 1 Institute for Soft Matter and Functional Materials, Helmholtz-Zentrum Berlin für Materialien und Energie GmbH, Albert-Einstein-Str. 15, 12489 Berlin, Germany; 2 ChIPS, University of Mons, B-7000, Mons, Belgium

**Keywords:** NEXAFS, STXM, TXM, X-ray microscopy

## Abstract

Today, in material science nanoscale structures are becoming more and more important. Not only for the further miniaturization of semiconductor devices like carbon nanotube based transistors, but also for newly developed efficient energy storage devices, gas sensors or catalytic systems nanoscale and functionalized materials have to be analysed. Therefore, analytical tools like near-edge X-ray absorption fine structure (NEXAFS) spectroscopy has to be applied on single nanostructures. Scanning transmission X-ray microscopes (STXM) as well as full-field transmission X-ray microscopes (TXM) allow the required spatial resolution to study individual nanostructures. In the soft X-ray energy range only STXM was used so far for NEXAFS studies. Due to its unique setup, the TXM operated by the Helmholtz-Zentrum Berlin (HZB) at the electron storage ring BESSY II is the first one in the soft X-ray range which can be used for NEXAFS spectroscopy studies which will be shown in this review. Here we will give an overview of the different microscopes used for NEXAFS studies and describe their advantages and disadvantages for different samples.

## Review

### Introduction

Several analysis tools and techniques have been developed over the last century to explore electronic and structural properties of materials. Laboratory-source based techniques have compared to methods performed at synchrotron radiation sources the disadvantage of less brilliant X-ray beams and are not tunable over a wide photon energy range. Therefore, with the availability of synchrotron radiation sources several techniques could be developed having smaller spot sizes which allows studies of the properties of small samples or details of larger materials. Protein crystallography is one of the widely used techniques becoming high productive at synchrotron sources. Macromolecular crystals of proteins, viruses or nuclic acids are studied at the atomic structural level [1]. The development of drugs by understanding the interaction is greatly influenced by this technique. X-ray microscopy techniques enable the study of thick (i.e., up to 10 µm) specimens in materials and biological sciences using photon energies that covers the K- and L-X-ray absorption edges of elements of major interest [2–5]. High-photon energy X-ray diffraction used in operando studies can correlate changes in size of the unit cell of catalysts with their deactivation [6]. Studies of the pore structure of rocks can be performed by using X-ray micro tomography. This allows visualizing and investigating changes of the 3D pore structure by injecting CO_2_ saturated brine [7–8]. The storage of CO_2_ in underground reservoir rocks is a promising approach to reduce the greenhouse gas emissions.

Parallel to the development of novel synchrotron sources, the nanoscale of the current technologies triggered the revision of X-ray absorption spectroscopy [9–10] or photoelectron spectroscopy [11–12] techniques. These techniques can be used to study the electronic structure of materials; however they probe typically areas of larger than 50 × 50 µm^2^. By applying them for nanostructures or nanoparticles the electronic structure information will be averaged over different individual nanostructures. To investigate the electronic structure of isolated nanostructures needs spatial resolution in the nanoscale range together with X-ray spectroscopy methods. X-ray microscopy reaches a higher Rayleigh resolution than optical microscopy as the resolution is decreasing linear with the wavelength. Additionally, the larger penetration depth and smaller radiation damage compared to electron microscopy has to be noted. Atmospheric pressure operation using a helium atmosphere is possible [13]. Electronic properties of samples can be studied due to the interaction of X-ray with matter. Therefore, the combination of X-ray microscopy and spectroscopy denominated X-ray spectromicroscopy having the capability of offering both spatial and chemical/physical information opens avenues for detailed characterization of nanostructures. Other spatially resolved techniques or spectromicroscopy as, e.g., electron energy loss spectroscopy (EELS) [14] have been chosen to study individual nanostructures/nanoparticles. By using monochromatic, aberration-corrected transmission electron microscopes (TEM) operating at low voltages spectromicroscopy of isolated nanostructures can be performed. Here, energy resolutions comparable to classical synchrotron based spectroscopy techniques can be achieved. A drawback of this technique is that it cannot handle samples which are thicker than a few atomic diameters [15–17]. By using linear polarized X-rays available at synchrotron radiation sources effects by dichroism in materials can be studied. Thus, in this case, the degree of alignment, molecular orientation as well as spectral assignments can be determined [9,18–20]. Such measurements of the polarization dependence (linear dichroism) relative to a characteristic direction of isotropic samples using the momentum transfer directional dependence in an electron microscope is possible, but highly affordable [21].

In the past, spectroscopic methods with high spatial resolution in the nanometer range were restricted to EELS microscopy [22–23] or scanning transmissions X-ray microscopes (STXM) [3,24]. These methods are well adapted to study the electronic structure of isolated nanostructures as their typical image fields contain only one or few nanostructures. Though STXMs operating at an undulator beamline allow polarization dependent studies it is time consuming to gather statistical information because of the sequential image formation process in the STXM: The focal spot of the objective is raster-scanned over the sample to form the image by detecting the transmitted X-ray photons. Additionally, only a small fraction of the spatially coherent undulator flux can be used whereas the recently reported HZB TXM operates with partial coherent X-rays from an undulator source and therefore, using a higher amount of the available flux [25].

### Soft X-ray microscopes

**Figure 1:**
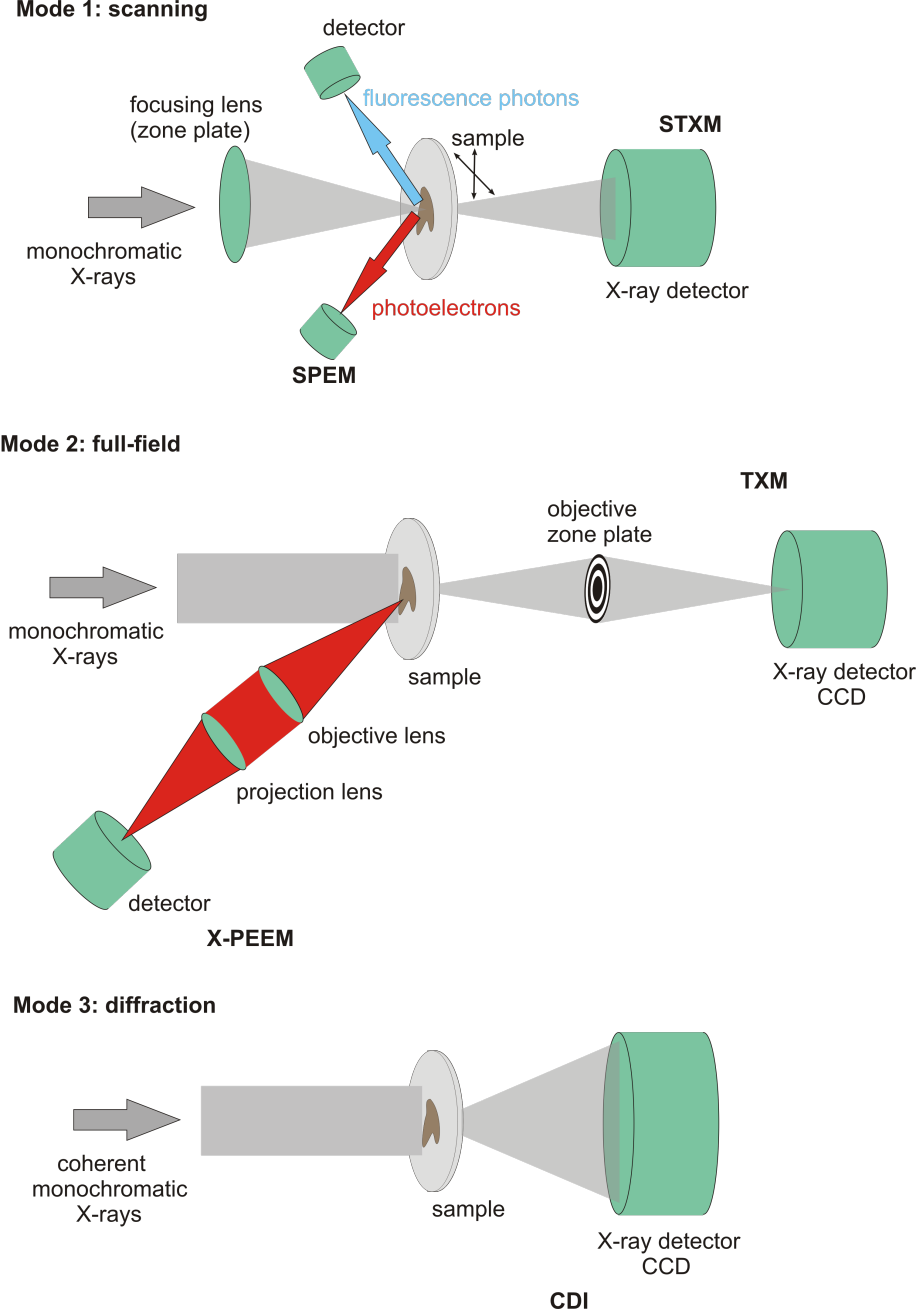
Schematic layout of general modes of soft X-ray microscopy. STXM and SPEM are focused probe methods, whereas TXM and X-PEEM are direct imaging methods. CDI needs computer algorithms to reconstruct an image from the recorded diffraction pattern.

In general three modes of soft X-ray microscopes can be distinguished [26] and are schematically illustrated in Figure 1:

**Mode 1 – scanning mode:** Producing an image by raster scanning the sample through a small focal spot generated by a focusing lens which is in most cases a zone plate (ZP). In a scanning transmission X-ray microscopy (STXM) the transmitted X-rays are detected. Scanning photoelectron microscopy (SPEM) is a method where the emitted photoelectrons are kinetic-energy-resolved detected. Additionally, fluorescence photons emitted from the sample can be detected.

**Mode 2 – full field mode:** Direct imaging of the sample using a zone plate objective is provided by transmission X-ray microscopy (TXM). In the past a condenser ZP has been used to illuminate the sample and an objective ZP projects a magnified image of the sample to an appropriate area detector. A similar direct imaging is available in X-ray photo-electron emission microscopy (X-PEEM) where monochromatic X-rays illuminate the sample. Here, electron optics, which can be electrostatic or magnetic lenses, are used producing a magnified image of the distribution of the ejected electrons on an electron sensitive camera.

**Mode 3 – diffraction mode:** Coherent diffraction imaging (CDI) methods use coherent monochromatic X-rays normally without any optical element to illuminate the sample. A suitable X-ray detector records the far-field coherent scattering signal. With the help of computer algorithms this pattern is inverted into a real space image. So far, samples with weak contrast are not easily reconstructed. NEXAFS possibilities are missing.

Nowadays, the spatial resolution for zone plate based X-ray microscopes reaches 10 nm for real, even having weak contrast, samples [27–29].

**Figure 2:**
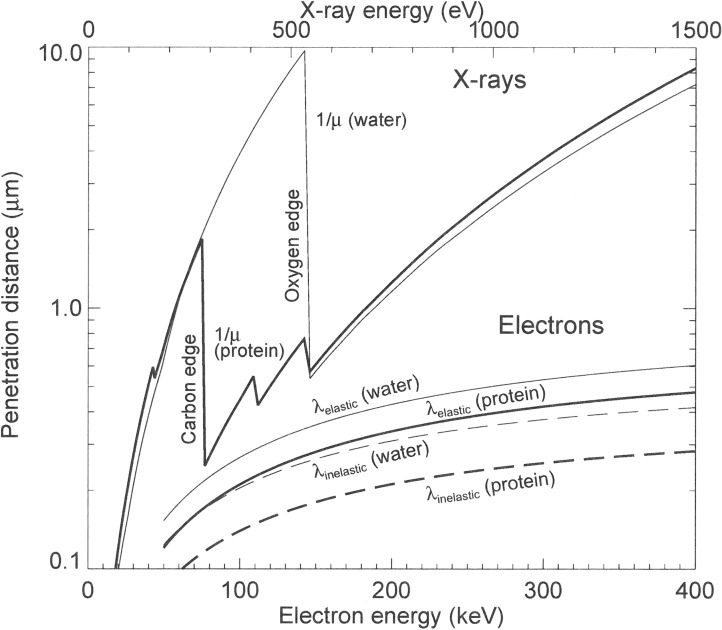
Calculated penetration depths of X-rays and electrons in dependence of their energy. The penetration depth of X-rays is larger than for electrons. In the so called “water window”, i.e., between the carbon and oxygen absorption edges at 284 and 543 eV protein and water have quite different absorption values which leads to a natural absorption contrast. Reprinted with permission from [30]. Copyright 1995 Cambridge University Press.

The information depth by using soft X-ray microscopes depends strongly on the applied method. For SPEM and X-PEEM electrons emitted from the sample surface are detected. These electrons are generated in the surface layer not deeper than 10 nm. STXM and TXM are bulk sensitive methods as they normally detect the photons which are transmitted through the sample. The penetration depth of X-rays and therefore the usable sample thickness correlates with the X-ray photon energy used for the analysis and is much larger than for electrons (Figure 2) [30]. In the soft X-ray regime penetration depths in the order of several microns are accessible. This allows probing buried nanostructures within a bulk sample. Note, that the interaction of electrons with matter is mainly determined through Coulomb forces, whereas the interaction of X-rays with matter is caused by the valence electron cloud which will give different information.

In this review only methods for bulk sensitive NEXAFS spectroscopy using STXM and TXM in the soft X-ray range will be further discussed.

**Figure 3:**
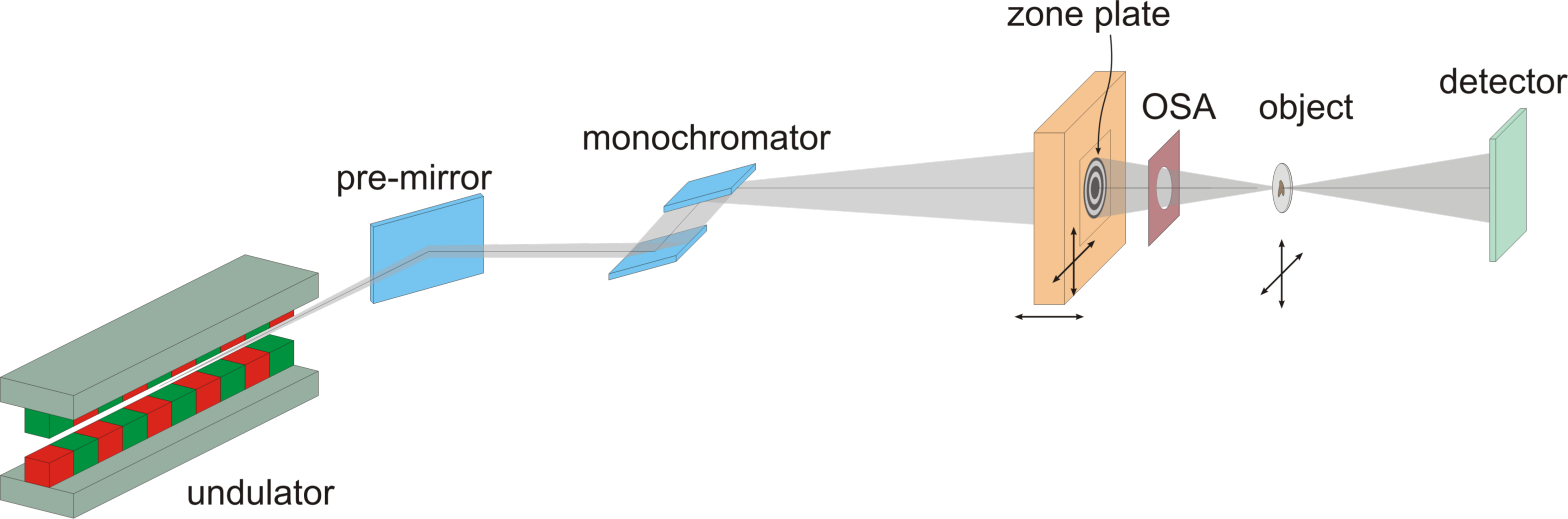
Schematic setup of a scanning transmission X-ray microscope (STXM).

The Stony Brook group at the National Synchrotron Light Source (NSLS) at Brookhaven National Laboratory built the first STXM having a zone plate lens to produce the scanning spot [31–32]. In order to get the diffraction limited resolution a STXM needs spatially coherent light. The schematic setup of most STXMs is shown in Figure 3: The coherent part of the X-ray beam passing through a monochromator is collected by a zone plate. The zone plate produces a diffraction limited spot in the sample plane. An order sorting aperture (OSA) filters the unwanted diffraction orders of the ZP. The sample is mounted on a stage having stepping or piezoelectric driven motors to perform the raster scan. The X-ray sensitive detector collects the transmitted X-ray photons. No low efficiency optic is upstream of the sample. Therefore, the radiation load to the sample is minimized. The pixel by pixel data format can be easily used for spectromicroscopy applications. In the STXM, the contrast transfer function (CTF) follows that one of an incoherent microscope due to the image formation process (pixel by pixel) and due to the fact that normally the detector aperture is equal or larger than that of the zone plate [33].

**Figure 4:**
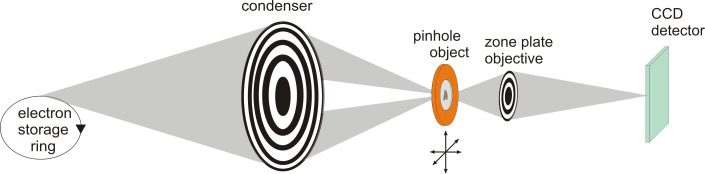
A full field transmission X-ray microscope (TXM) for a bending magnet source uses two zone plate lenses, condenser and objective, to form a 2D image on the detector. Additionally, a pinhole close to the sample is necessary which acts together with the condenser as a monochromator.

The first TXM having a higher resolution than light microscopes was constructed by the group of Günter Schmahl, University of Göttingen at the electron storage ring ACO in Orsay, France [34–35]. Figure 4 shows the set-up of a typical TXM at a bending magnet source. In similarity to a conventional light microscope the electron storage ring is used as the light source. The condenser zone plate collects the incoming X-rays and is used for the illumination of the sample. A high resolution zone plate objective generates a magnified image of the illuminated sample area on the X-ray sensitive CCD camera. A pinhole is required between the condenser and the sample which acts together with the condenser zone plate as monochromator. Additionally, a central stop near the condenser zone plate is used to block direct light and to produce a hollow cone illumination.

Depending on the available photon flux from the source, the efficiency of the zone plate objective and the sensitivity of the CCD detector exposure times in the range of a few seconds for acquiring a full-field image are state of the art. TXMs are not requiring precise scanning stages as STXMs and do not need spatially coherent illumination. So far, large diameter zone plates [36] or multilayer mirrors [37–38] were used as condenser. The drawback of these condenser types is their limited monochromaticity (*E*/Δ*E*) in the range of a few hundred hindering spectroscopic applications. The full field TXM at the undulator beamline U41-FSGM at the electron storage ring BESSY II operated by the Helmholtz-Zentrum Berlin (HZB) uses a different optical concept [25]: The spectral resolution needed for NEXAFS studies is provided by the monochromator in front of the condenser. Therefore, a spectral resolution *E*/Δ*E* in the range of 5000 which is comparable to STXM setups can be reached. The achromatic condenser is a novel ellipsoidal shaped capillary which has an efficiency one order of magnitude higher than ZPs [39]. Due to the setup at an undulator beamline and the smaller size of the condenser compared to a ZP condenser partial coherent X-rays illuminates the condenser. The maximum possible resolution in this partial coherent case is lower compared to the incoherent case, but the contrast transfer function (CTF) shows higher contrast values at medium to higher spatial frequencies [25,29]. Combining partial coherence with a high-resolution objective, the capillary optic of the HZB TXM leads to high contrast C_object_ for nanoscale features which reduces artefacts of the measured absorption values. The radiation dose for detecting small object features is proportional to (C_object_ ∙ CTF)^−2^ [29,40]. As a consequence of this favourable shape of the contrast transfer function exposure time and sample damage are minimized.

### Results and Discussion

Today, several STXMs in the soft X-ray region are in operation at many synchrotron light sources worldwide [41]. Here, we restrict ourselves to a short overview of the most used instruments by the scientific community: At the Advanced Light Source (ALS) in Berkeley, California, USA, two STXMs [42–44] are operated for polymer sciences [45] and for the investigation of magnetic nanostructures [46–47]. In the latter case the magnetic properties as a function of an applied magnetic field as well as time resolved measurements [48] are performed to study the electronic functionalities for example in magnetic random access memories (MRAMS) or hard discs. The Hitchcock team of the McMaster University operates a STXM at an undulator beamline at the Canadian Light Source (CLS), Saskatoon, Canada, for many applications as, e.g., polymer sciences at the carbon K-edge [49], studies on graphene [50] and investigations of biological materials [51]. The STXM “Pollux” [52–53] installed at the Swiss Light Source operated by the Paul Scherrer Institute, Villingen, Switzerland, is a versatile instrument also covering polymer and magnetic investigations [54–55]. “Maxymus” (Magnetic X-ray Micro- and UHV Spectroscope) at the BESSY II electron storage ring, Helmholtz-Zentrum Berlin, Germany, is operated by the Max-Planck-Institut for Intelligent Systems, Stuttgart, Germany and allows – compared to other STXMs – investigations under ultrahigh vacuum conditions [56]. This instrument is dedicated for studies of the magnetic behaviour of solids in the nanoscale range and allows time resolved measurements [57].

**Figure 5:**
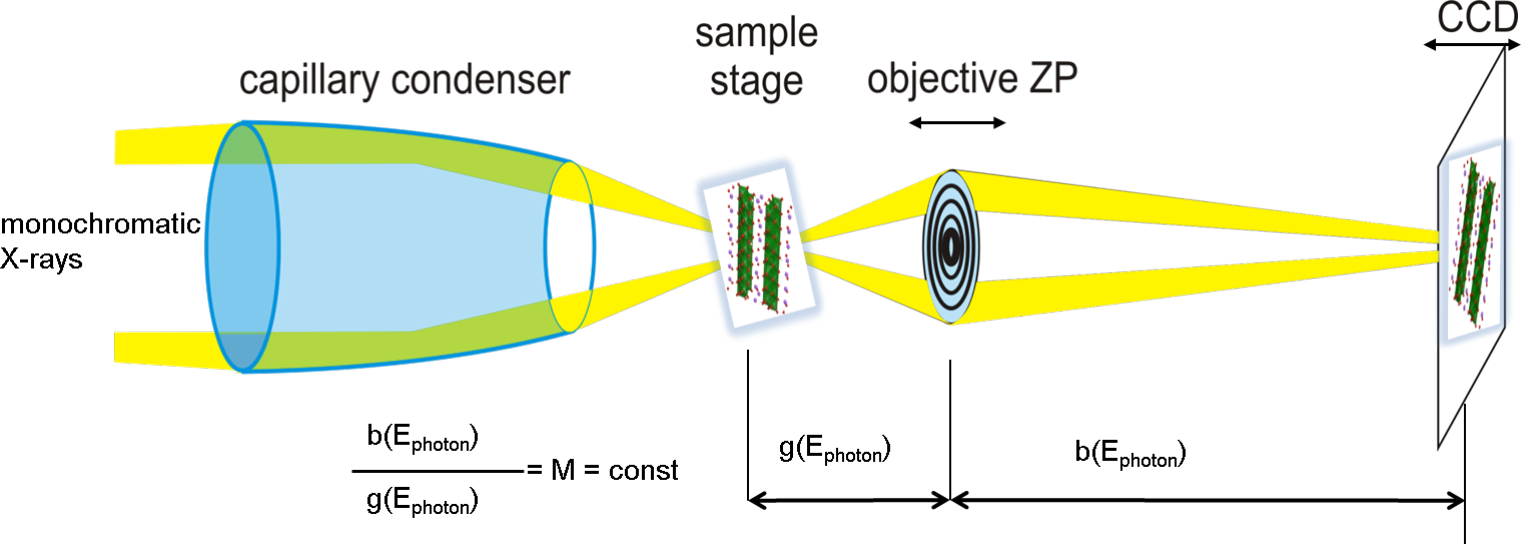
Schematic setup of the HZB-TXM for NEXAFS studies: Monochromatic X-rays are collected with an achromatic capillary condenser to illuminate the sample. At each photon energy a highly magnified 2D image of the sample is taken whereupon the magnification (M) is kept constant [58].

With the new optical concept of the HZB-TXM it was demonstrated for the first time that NEXAFS by studying sodium titanate nanostructures at the Ti-L-absorption edge in the soft X-ray region can be performed with a full field microscope (Figure 5) [58]. It should be noted that the collection rate for similar field of views is about two orders of magnitude faster than is possible with STXMs. By combining multichannel multiple-scattering calculations with the NEXAFS-TXM data specific spectral features at the Ti-L-edge could be associated to titanium atoms in distinct atomic sites within the lattice [58–59].

After these first encouraging investigations further spectroscopic studies on nanostructures containing metal atoms like copper were performed. It was shown that chemical analysis of nanowires for solar cells can be performed by NEXAFS-TXM. As a result of this investigation it was found that neither Cd nor S diffuse into the CuO phase after the deposition of CdS on the CuO nanowires [60–61]. The possibility to study the oxidation state of Mn dopants in titanate nanostructures was reported in [62].

Additionally, using the capability to investigate cryogenic samples in the HZB-TXM, the electronic structure of individual hybrid colloid particles in their hydrated environment were analysed [63]. Here, the structural homogeneity of nanoparticles in the hybrid particle was examined. Nanoscale valence changes in resistive switching thin film devices (SrTiO_3_) could be demonstrated [64]. The change of resistance in a RRAM device could be assigned to a redox-process. The switching filament could be allocated to extended growth defects which are already present in the virgin films. Synthesis of anisotropic core-shell Fe_3_O_4_@Au magnetic nanoparticles which will have future applications in photo thermal therapy or drug delivery can be optimized by different analysis methods including NEXAFS spectroscopy with the HZB-TXM [65]. In the latter case as well as in the case of hybrid colloid particles the nanoparticles have sizes below the spatial resolution of the TXM, but still provide signals which can be used for NEXAFS spectroscopy.

**Figure 6:**
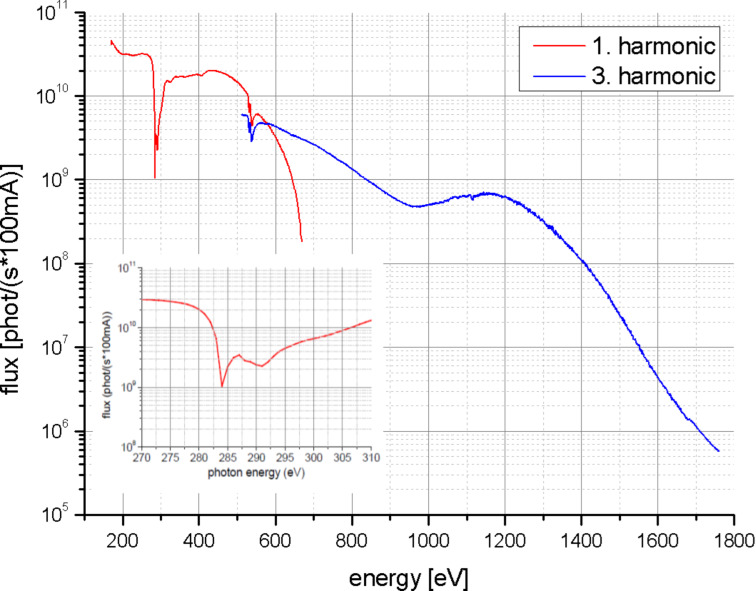
Measured integrated photon flux at the sample position of the HZB-TXM using a 10 µm exit slit of the monochromator for the 1. and 3. harmonic of the undulator. The inset shows the situation at the carbon-K-edge: A decrease of the flux due to the carbon contamination of the optical elements and the effect of the second order radiation (double photon energy, without using any filter).

Spectroscopic studies at the carbon K-edge are very challenging because nearly in all beamlines the photon flux is sharply reduced within the carbon K-edge photon energy range (280 up to 320 eV) due to the absorption of the X-ray beam by a thin carbon film contamination deposited on the optical elements (grating, mirrors, objectives). This contamination is caused by the interaction of the X-ray beam with residual hydrocarbon molecules in the vacuum of the beamline. The integrated photon flux at the sample position in the U41-FSGM beamline at the electron storage ring BESSY II (Figure 6) is reduced by one order of magnitude and, in addition, a peak related to second harmonic radiation (double photon energy) exists in the carbon K-edge energy region. Suppression of these higher energies can be accomplished by the use of appropriate filters. In the case of the carbon K-edge normally Ti-filters are in use. The sharp decrease in the photon flux at the photon energy range of the carbon K-edge add difficulties in the analysis of the carbon nanostructure due to the low contrast images recorded in this region. It is important to mention that the study of the carbon K-edge is also a challenge in non-microscopic NEXAFS set-ups due to uncertainties in the normalization by the photon flux. Nevertheless, carbon nanostructures, like suspended carbon nanohorns (CNH) [66], or thin graphite sheets [2] could be studied with the HZB-TXM. The electronic states of freestanding CNH aggregate in a dahlia-like shape were investigated and could be related to the presence of pentagonal rings and folding of the graphene sheet in the CNH [66]. Metal impurities due to the exfoliation process of graphite give rise to a pre-edge signal at 284.2 eV. Topological defects added additional spectral features in the π and σ region of the absorption edge [2].

STXMs have no optical element between the sample and the detector. Therefore, the radiation dose applied to the sample during the investigation might be lower. This is helpful in the case of very radiation sensitive materials like some polymers [45] which cannot be analysed in the TXM. Cryo preservation can be used for the structural integrity of the sample [67] but even at these low temperatures atomic bindings of sensible materials are damaged, i.e., the electronic structure is changed. The resulting STXM spectra of those sensitive materials have to be checked very carefully to avoid misinterpretations due to possible radiation induced changes.

Some interesting nanostructure materials like boron nitride could not be investigated so far at the boron-K-edge with the HZB-TXM, as no adapted objective with a longer focal length is available. However, such NEXAFS studies were performed with the STXM at the Canadian Light Source (CLS, Saskatoon, SK, Canada) [68].

### Experimental

**Figure 7:**
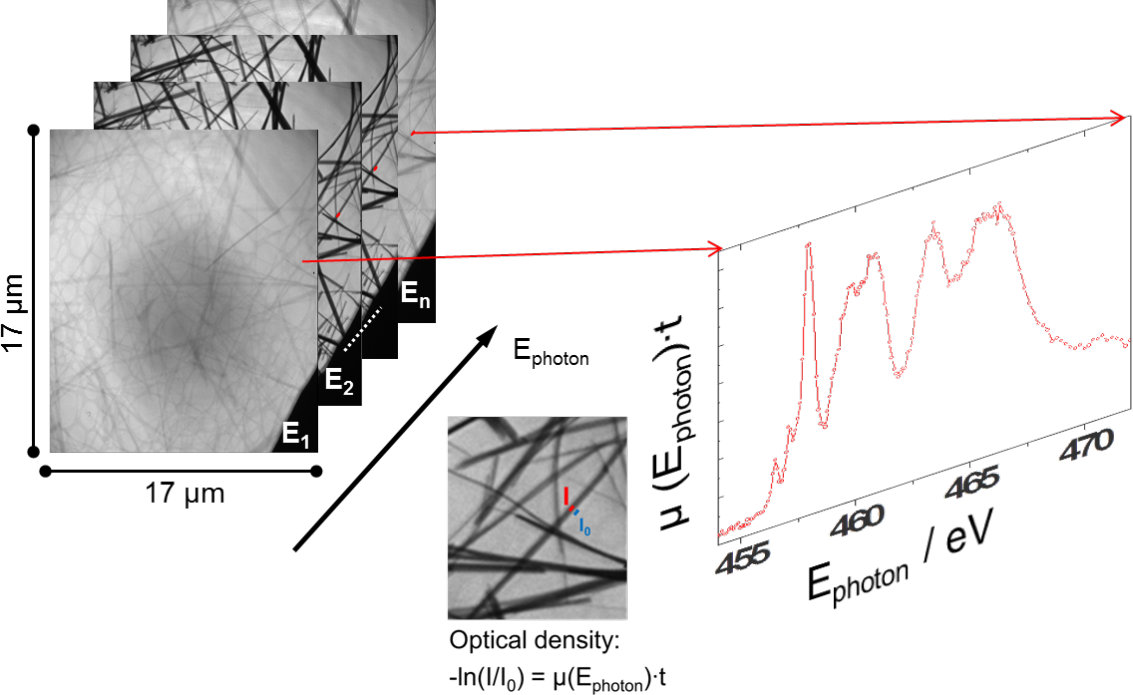
Workflow for NEXAFS-TXM: A data set of images at different photon energies is taken with the HZB-TXM. A NEXAFS spectrum can be generated out of this data set by using defined areas of the sample to measure *I*(*E*) and neighbouring blank areas for *I*_0_(*E*) [58].

For NEXAFS spectroscopy, it is necessary to measure one data set for all photon energies *E* of the transmitted intensity *I*(*E*) through the sample and another data set *I*_0_(*E*) without sample. The absorption spectrum normally will be displayed as an optical density OD(*E*) = −log[*I*(*E*)/*I*_0_(*E*)] [69]. So far, nearly all absorption spectroscopy techniques cannot measure *I*(*E*) and *I*_0_(*E*) at the same time which can lead to errors in the normalization due to instabilities of the photon beam. For nanostructures the photon flux (*I*_0_(*E*)) and the signal (*I*(*E*)) can be recorded in the full field HZB TXM simultaneously at near the same position reducing the uncertainties in the normalization of the NEXAFS signal. For this, near the nanostructure we should have a bare region (or a hole) where the *I*_0_(*E*) can be extracted (Figure 7). The analysis software package aXis2000 [66] which was originally written for STXM data analysis can now also handle the HZB TXM data sets.

### Conclusion

Bulk sensitive nanoscale NEXAFS spectroscopy was restricted in the past to STXMs as TXMs did not allow the necessary spectral resolution. With the recently developed setup of the HZB-TXM at the BESSY electron storage ring this restriction could be overcome. As shown in this review, several published NEXAFS-TXM studies of low dimensional nanostructures pave the way for understanding the electronic structure towards the atomic scale and will help in the design of tailored functional systems. The investigated carbon nanotubes, nanowires as well as nanoscale oxides have due to their high surface to volume ratio an enormous potential in nanoelectronics, catalysis, light harvesting and energy storage applications. The newly developed NEXAFS-TXM together with further complementary physicochemical methods and spectroscopic techniques will allow a more complete understanding of the function of low dimensional nanostructures.
